# Fox on the run – molecular surveillance of fox blood and tissue for the occurrence of tick-borne pathogens in Austria

**DOI:** 10.1186/s13071-014-0521-7

**Published:** 2014-11-21

**Authors:** Georg Gerhard Duscher, Hans-Peter Fuehrer, Anna Kübber-Heiss

**Affiliations:** Institute of Parasitology, Department of Pathobiology, University of Veterinary Medicine Vienna, Veterinaerplatz 1, Vienna, A-1210 Austria; Research Institute of Wildlife Ecology, University of Veterinary Medicine, Vienna, Austria

**Keywords:** *Hepatozoon canis*, *Anaplasma phagocytophilum*, *Babesia microti*-like

## Abstract

**Background:**

The red fox (*Vulpes vulpes*) is a widespread species, harbouring many pathogens relevant for humans and pets. Indeed, *Anaplasma* spp., *Ehrlichia canis* and *Rickettsia* spp. among the bacteria and *Hepatozoon canis* as well as *Babesia* sp. among the parasites have been the focus of several studies.

**Findings:**

In a cohort of 36 foxes shot on one day in the north-eastern part of Austria, *Babesia microti*-like pathogens were found in 50%, while *H. canis* was detected in 58.3% of the samples. The spleen was more useful for detection of *H. canis*, whereas *B. microti*-like parasites were more frequently found in the blood. Bacteria could not be confirmed in any of the cases to demonstrate the occurrence of such tick-borne pathogens using PCR and sequencing on blood and spleen samples.

**Conclusions:**

The occurrence of *B. microti*-like and *H. canis* parasites raised many questions, because these infections have never been found autochthonously in dogs. Furthermore in the case of *H. canis* the main vector tick, *Rhipicephalus sanguineus*, is absent in the sampling area, leaving space for further hypotheses for transmission such as vertical transmission, transmission via ingestion of prey animals or other vector ticks. Further studies are needed to evaluate the risks for pets in this area. PCRs delivered differing results with the different tissues, suggesting the use of both spleen and blood to obtain an integral result.

## Findings

### Background

Red foxes (*Vulpes vulpes*) are among the most widely distributed mammals in the world and are invading many urban areas due to a good adaptation to human environments, and to rabies vaccination [1]. As a result foxes might play a big role in spreading pet-relevant pathogens and parasites such as mites and ticks [2]. Recently they have been discussed as a potential reservoir for blood parasites like *Anaplasma phagocytophilum* [3], *Hepatozoon canis* [4], *Babesia* sp. [5], *Ehrlichia canis* [6] and *Rickettsia* spp. [2]. Due to their close vicinity to domestic habitats they may act as a transmission interface for some of these pathogens to pets and humans [5].

*Babesia microti*-like parasites – also known as *Babesia* sp., *Babesia annae* or *Theileria annae* – are frequently found in foxes in countries such as Croatia [7], Portugal [5] and Spain [8]. The common assumption is that *Ixodes hexagonus* is involved in the transmission cycle [9], and a recent study identified *I. ricinus* and *I. canisuga* as carriers and therefore as potential vectors [10]. These ticks could also serve as a transmission interface to dogs, where *Babesia* may cause azotaemia, haemolytic anaemia, renal failure and mortality [11].

*Hepatozoon canis* affects canids and its occurrence is mostly linked to the distribution of the main vector tick *Rhipicephalus sanguineus* [12], already displaying exceptions in countries such as Austria, Germany or Hungary [12–14].

The aim of this study is to evaluate the role of foxes in terms of their blood pathogens and to discover potential reservoirs for tick-borne diseases in northern latitudes.

## Method

Foxes shot on 18 January 2014 in the district of Gänserndorf (in the northeast of Lower Austria) were further processed on the same day. From the 36 foxes, 35 spleen samples and 17 blood samples were obtained. Extraction of DNA from blood and tissue was performed as previously described [14]. Primers detecting *Anaplasma* sp., *Babesia* sp. (piroplasms), *Ehrlichia canis, Hepatozoon canis* and *Rickettsia* sp. were used (Table 1). The PCRs were conducted on the Eppendorf Mastercycler pro S (Eppendorf AG, Hamburg, Germany) using protocols published elsewhere [14]. To confirm the sequence, positive samples were randomly chosen and the amplifications were purified by Fast-kit (Bio-Rad Laboratories, Vienna, Austria) according to the manufacturer’s recommendations and sent for sequencing (Microsynth AG, Balgach, Switzerland; LGC, Teddington, UK). Sequences obtained were further processed by GeneDoc (http://genedoc.software.informer.com/2.7/) and blasted on GenBank® (http://blast.ncbi.nlm.nih.gov/Blast.cgi).

**Table 1:** **PCR parameters for amplification of DNA of target organisms**

**Target organism**	**Forward primer (5’-3’)**	**No. of cycles**	**Annealing temperature (°C)**	**Primer concentration (pmol)**	**Product size (bp)**	**Reference**
	**Reverse primer (5’-3’)**					
*Anaplasma* sp.	Ehr.u.for: GTT TGA TCC TGG CTC AGG AYD AAC	30	66.8	12.5	619	[15]
	ERB2rev: CTC TTT CGA CCT CTA GTC TAG C					
Piroplasms (nested)	1st	40	68	25	561	[16]
	BTH-1 F: CCT GAG AAA CGG CTA CCA CAT CT					
	BTH-1R: TTG CGA CCA TAC TCC CCC CA					
	2nd					
	GF2: GTC TTG TAA TTG GAA TGA TGG	40	60	50		
	GR2: CCA AAG ACT TTG ATT TCT CTC					
*Ehrlichia canis*	Ehr.u.for: GTT TGA TCC TGG CTC AGG AYD AAC	30	65.0	20	619	[15]
	Ehr.CCE.rev: CTC TTT CGA CCT CTA GTC TAG C					
*Hepatozoon canis*	HEPF: ATA CAT GAG CAA AAT CTC AAC	35	57.0	10	660	[17]
	HEPR: CTT ATT ATT CCA TGC TGC AG					
*Rickettsia* sp.	ITS-F: GAT AGG TCG GGT GTG GAA G	35	52	1	342 – 533	[18]
	ITS-R: TCG GGA TGG GAT CGT GTG					

### Ethical statement

Fox were shot during routine hunting events under the restrictions of the game laws of the province of Lower Austria.

## Results

The investigation of the blood and spleen samples identified 18 *B. microti*-like pathogen-positive foxes, 21 foxes harbouring *H. canis* and four foxes with double infections (Table 2), leading to prevalences of 50%, 58.3% and 11.1%, respectively. PCRs for detecting piroplasms (*Babesia sp.* nested) in blood and spleen detected 13 (76.5% of the blood samples) and 11 (31.4% of the spleens) *B. microti*-like pathogens, respectively. Sequences of these pathogens showed 98–100% similarity to *B*. sp. “Spanish dog” (e.g. GenBank® accession no. AF188001.1 or EU583387.1). Using the *Hepatozoon-*specific primers, 21 foxes tested positive for *H. canis*. The investigation of the spleen samples identified 18 positive results (51.4%), whereas in the blood samples only six positive results (35.3%) were found. Seven more PCR products, positive on the gel, provided no conclusive sequence data, and therefore were noted as false positives. All conclusive sequences delivered 99–100% similarity to *H. canis* found in GenBank® (e.g. accession no. AY150067.2, DQ111754.1, JN584477.1 or KC509526.1).

**Table 2:** **PCR results of spleen and blood compared to sequencing results of the investigated foxes (pos = representing a positive PCR product on the gel, neg = delivering no band on the gel,** ***H.canis*** **or** ***B. microti*** **-like = confirmed sequence of this pathogen in the substrate, “f” indicates false positive samples showing a gel band, but not confirmed during sequencing)**

	**PCR**			
**Fox**	***Piroplasms*** **nested**	***H. canis***	**Pathogens detected**	**GenBank® accession no**
1	*B. microti*-like	pos. ^f^	*B. microti*-like	KM115968
2	*H. canis*	*H. canis*	*H. canis*	KM115969
3	*H. canis*	pos.	*H. canis*	KM115970
4	*H. canis*	*H. canis*	*H. canis*	KM115971
5	*B. microti*-like	neg.	*B. microti*-like	KM115972
6	*B. microti*-like	pos. ^f^	*B. microti*-like	KM115973
7	*H. canis*	*H. canis*	*H. canis*	KM115974
8	*B. microti*-like	neg.	*B. microti*-like	KM115975
9	*B. microti*-like	neg.	*B. microti*-like	KM115976
10	pos	pos. ^f^	unclear	
11	*B. microti*-like	pos. ^f^	*B. microti*-like	KM115977
12	*B. microti*-like	neg.	*B. microti*-like	KM115978
13	*H. canis*	*H. canis*	*H. canis*	KM115979
14	*B. microti*-like	*H. canis*	*B. microti*-like/*H. canis*	KM115980/KM115981
15	*B. microti*-like/ *H. canis*	pos.	*B. microti*-like/*H. canis*	KM115982/KM115983
16	*H. canis*	*H. canis*	*H. canis*	KM115984
17	*B. microti*-like	pos. ^f^	*B. microti*-like	KM115985
18	*H. canis*	*H. canis*	*H. canis*	KM115986
19	*H. canis*	*H. canis*	*H. canis*	KM115987
20	*B. microti*-like	*H. canis*	*B. microti*-like/*H. canis*	KM115988/KM115989
21	*B. microti*-like	neg.	*B. microti*-like	KM115990
22	*H. canis*	*H. canis*	*H. canis*	KM115991
23	*B. microti*-like	neg.	*B. microti*-like	KM115992
24	*H. canis*	*H. canis*	*H. canis*	KM115993
25	*B. microti*-like	neg.	*B. microti*-like	KM115994
26	*H. canis*	*H. canis*	*H. canis*	KM115995
27	*H. canis*	*H. canis*	*H .canis*	KM115996
28	*B. microti*-like	pos. ^f^	*B. microti*-like	KM115997
29	*H. canis*	*H. canis*	*H. canis*	KM115998
30	*B. microti*-like	*H. canis*	*B. microti*-like/*H. canis*	KM115999/KM116000
31	*H. canis*	*H. canis*	*H. canis*	KM116001
32	pos.	*H. canis*	*H. canis*	KM116002
33	*H. canis*	*H. canis*	*H. canis*	KM116003
34	*B. microti*-like	neg.	*B. microti*-like	KM116004
35	*H. canis*	*H. canis*	*H. canis*	KM116005
36	*B. microti*-like	pos. ^f^	*B. microti*-like	KM116006

In none of the blood or spleen samples could *Anaplasma* sp., *E. canis* or *Rickettsia* spp. be detected.

## Discussion

Foxes are known to be major reservoirs for *Babesia microti*-like parasites [5]. The high prevalence of 50% found in this study and in this population is therefore not surprising and reflects a similar situation in Germany with 46.4% [10], Portugal with 69.2% [5] and Spain with 14% to 50% [8].

The 58.3% positive *H. canis* foxes in Austria are in concordance with four positive foxes out of nine found in Slovakia [19], 45.2% in Germany [20], 16 out of 111 investigated foxes (11.6%) in Poland [21] or 8% in Hungary [22]. To date *H. canis* is not found endemically in dogs in these areas, nor is *R. sanguineus* known to occur autochthonously [12,19,21,23], although *H. canis* has already been found in dogs in areas lacking the main vector tick in Germany [13,20] and Hungary [12,22].

## Conclusion

Foxes represent a good reservoir for several zoonotic and pet-relevant diseases. In terms of blood parasites this seems more the rule than the exception. Human- and pet-relevant agents such as *Babesia microti*-like pathogens and *H. canis* could be found in a relatively small set of fox samples originating from north-eastern Austria. Especially, the occurrence of *H. canis* in considerable numbers in this population so far north raises many questions such as the potential impact on domestic animals, reservoirs and infection pathways. Moreover, the main vector tick, *Rhipicephalus sanguineus*, is absent in the sampling area. Therefore other transmission pathways such as vertical transmission, transmission via ingestion of preyed animals or other vector ticks need to be evaluated.

Thus foxes have to be considered during treatment strategies and *B. microti-*like as well as *H. canis* pathogens have to be recognized as an unnoticed threat in northern areas. The use of piroplasm PCRs could help to identify both *B. microti*-like and *H. canis* pathogens prior to screening, followed by PCRs with species-specific primers.
